# Clozapine use at a specialised psychiatric hospital in Johannesburg

**DOI:** 10.4102/sajpsychiatry.v29i0.1999

**Published:** 2023-04-04

**Authors:** Katherine L. Ord, Belinda Marais

**Affiliations:** 1 Department of Psychiatry, Faculty of Health Sciences, University of the Witwatersrand, Johannesburg, South Africa

**Keywords:** clozapine, treatment-resistance, schizophrenia, side effect, treatment discontinuation, polypharmacy, South Africa

## Abstract

**Background:**

Clozapine is the gold standard medication for treatment-resistant psychosis, with robust evidence supporting its efficacy in multiple symptom domains. However, clozapine’s side effect profile contributes to its underutilisation and discontinuation.

**Aim:**

This study aimed to explore the magnitude of clozapine use and describe factors that impact on its effective use among in-patients.

**Setting:**

Tara Hospital, a specialised psychiatric hospital in Johannesburg.

**Methods:**

This was a retrospective, cross-sectional file review of clozapine-treated patients admitted over the 2-year study period. Data variables included: demographics, clinical information, discharge prescription, clozapine-related side effects and details of clozapine discontinuation, where applicable.

**Results:**

A cohort of 33.2% of patients from Tara’s biological wards received a trial of clozapine. Participants experienced anti-cholinergic clozapine-related side effects that included weight gain (79.5%), tachycardia (35.2%) and constipation (35.2%). Clozapine was discontinued in 13.7% of participants, and no life-threatening side effects or deaths occurred. Significantly more use of flupenthixol decanoate (64.3% vs. 30.7%; *p* = 0.0322) and anticholinergics (35.7% vs. 11.4%; *p* = 0.0474) occurred in the clozapine-discontinued group. Polypharmacy rates were high for psychiatric and non-psychiatric medications.

**Conclusion:**

One-third of patients received clozapine trials, most of whom continued at discharge. Although side effects occurred frequently, life-threatening side effects did not. Clozapine monitoring protocols, side effect rating scales, pre-emptive management of side effects, lifestyle interventions and clinician education may improve outcomes of clozapine use. The use of plasma clozapine levels may be beneficial.

**Contribution:**

This study expands our limited knowledge regarding current clozapine prescribing trends in South Africa.

## Introduction

Clozapine is the only evidence-based antipsychotic indicated for treatment-resistant psychosis.^1^ Clozapine has proven efficacy in the reduction of recurrent suicidal ideation in patients with schizophrenia and schizoaffective disorder.^2^ A study of the death registers in Finland, spanning from 1996 to 2006, indicated that their clozapine-prescribed population had overall lower all-cause mortality rates when compared with the population on alternative antipsychotics. This benefit was attributed to clozapine’s reduction in suicidality.^3^ Clozapine is indicated for the management of tardive dyskinesia,^2^ and has proven efficacy in treating anxiety, irritability, aggression and negative symptoms of schizophrenia,^4^ and benefits in cognitive functioning.^5^

Clozapine has an extensive receptor binding and pharmacological profile, responsible for both its therapeutic efficiency and wide range of potential side effects.^6^ With some exceptions, most clozapine-related side effects occur within 3 to 6 months of beginning treatment. Rarely clozapine discontinuation may be required for severe or life-threatening side effects. Indications for discontinuation, without re-challenge, include agranulocytosis (absolute neutrophil count [ANC] < 0.5 × 10^9^/L)^7^ myocarditis, cardiomyopathy and prolonged cardiac ventricular repolarisation (QTc) interval (> 500 ms). Ileus, neuroleptic malignant syndrome, venous thromboembolism and hyperglycaemic emergencies are also side effects requiring clozapine discontinuation. However, in such cases, once the patient is medically stabilized, clozapine may be re-challenged. Discontinuation of clozapine however does increase the risk of relapse, aggression and suicidality, and as such careful consideration is taken when planning intervention in the management of side effects.^8^ Most clozapine-related side effects do not warrant discontinuation and commonly attenuate with time or dose reduction.^9^ In a recent systematic quantitative meta-review, clozapine proved to have superior outcomes with regard to hospitalisations, discontinuation rates and mortality when compared with other antipsychotics.^10^

Regarding clozapine’s more commonly occurring side effects, prevalence rates of metabolic syndrome range between 11% and 64% in clozapine users, compared with a prevalence of 20% in first-episode schizophrenia patients, pre-treatment. Weight gain is experienced in up to 35% of patients, at an average of 4.4 kg over 10 weeks. Gastrointestinal side effects include hypersalivation, constipation, gastrointestinal reflux disease, nausea and vomiting, and occur more frequently with clozapine compared with other antipsychotics. Tachycardia is the most common cardiac side effect, with a prevalence of 25%–35%. The historically synonymous clozapine-related side effect, neutropenia, however, has an overall risk of about 3%.^9^

Many of clozapine’s side effects are dose-dependent, yet because of significant inter-individual variation in clozapine metabolism, as well as drug–drug interactions,^11^ some patients experience side effects at much lower doses than others. Clozapine is metabolised by the hepatic cytochrome P-450 isoenzyme system, specifically CYP1A2 and CYP3A4, and has active metabolites, norclozapine and clozapine-N-oxide. It has an extensive list of pharmacokinetic drug interactions, resulting in inhibition and induction of this system and altering the plasma concentration of the active metabolites. Consequently, co-administered medications may affect clozapine plasma concentration. Furthermore, certain genetic polymorphisms of cytochrome enzymes are associated with changes in clozapine clearance. Other factors such as gender, age and cigarette smoking can also affect the pharmacokinetics, and thus plasma concentration of clozapine.^12,13^ Therapeutic drug monitoring is a tool used in some settings to optimise pharmacotherapy,^14^ but is not currently routinely performed in South Africa (SA).

Although the side effect burden of clozapine is high, side effects can generally be managed by dose reduction, dose-splitting or with pharmacological intervention.^15^ A recent audit performed in the United Kingdom (UK) indicated that two-thirds of patients on clozapine were on further medications to manage side effects.^16^ Regarding polypharmacy in schizophrenia, aside from medications added to manage side effects or comorbid diseases, patients may also be treated with multiple psychotropic medications. Antipsychotic polypharmacy is concerning because of the lack of standardised research describing efficacy and safety, and unclear practice patterns.^17^ The benefit of polypharmacy must always outweigh the risks,^17^ which include increased pill burden, increased risk of side effects and drug–drug interactions, increased risk for accidental medication errors, or non-adherence by virtue of complicated treatment schedules, as well as higher economic expense.^18^ Although polypharmacy should generally be avoided, in the case of clozapine, augmentation strategies and combination therapies may improve patient outcomes,^18^ and minimise the need for clozapine discontinuation. Despite clozapine monotherapy being strongly advised,^18^ evidence to support the so-called ‘clozapine combination therapy’ is growing.^15^

With regard to patterns of clozapine use worldwide, despite clozapine being well-recognised as the gold standard antipsychotic, a retrospective study performed in the United States of America (USA) from 2002 to 2005 indicated clozapine prescription rates were low for the population of patients who met criteria for clozapine trial.^19^ In the 2018–2019 National Clinical Audit of Psychosis in the UK, it was found that only 54% of patients meeting the criteria for treatment resistance were offered clozapine. Based on this finding, the Healthcare Quality Improvement Partnership recommends that reasons for not prescribing clozapine be documented in records and advised that mental health pharmacists should assist with identifying patients who are eligible for clozapine.^1^ A cohort study performed in Denmark assessing the global assessment of the functioning of patients initiated on clozapine between 2004 and 2011 found that there was moderate to substantial functional improvement in more than 24% of clozapine users, but that chance of substantial improvement in females decreased by 15% for each year that clozapine initiation was delayed.^20^

Clozapine is broadly underutilised and, based on evidence to support its efficacy, this is a public health dilemma. While a study on international clozapine trends across 17 countries found that clozapine prescription is on the increase, the countries that were included were high-income, and there remains little data on clozapine use in the third world.^21^ Compounding the aforementioned, there is limited research describing the landscape of psychiatric illness and the use of clozapine in our setting. In a study performed in the Western Cape, clozapine use in Xhosa-speaking patients diagnosed with schizophrenia or schizoaffective disorder was low, at 10%, with a rate of antipsychotic polypharmacy almost three times higher, at 28%.^22^ Small studies on clozapine users have described the prevalence of clozapine-related side effects,^23^ and monitoring patterns of patients on clozapine.^24^ However, research describing clozapine prescribing patterns in SA is sparse.

## Aim and objectives

This study aimed to explore the magnitude of clozapine use, and the demographic, clinical and prescription characteristics of inpatients treated with clozapine, at a specialised psychiatric hospital in Johannesburg, SA.

The study objectives were:

- To determine the magnitude of, and indication for, clozapine use among patients admitted to the biological wards at Tara Hospital, over a 2-year period, from 01 January 2018 until 31 December 2019
- To determine patient demographic, clinical and prescription characteristics of the patients treated with clozapine at Tara Hospital during the study period
- To determine the prevalence of clozapine-related side effects
- To determine the rate of clozapine discontinuation without rechallenge during the admission. Furthermore, in those who discontinued clozapine, to describe the reason for discontinuation.

## Research methods and design

### Study design and setting

This study was a retrospective, cross-sectional file review conducted at Tara Hospital, which is a government sector specialised psychiatric hospital in Johannesburg. Tara Hospital offers outpatient and inpatient psychiatric services, with a total bed capacity of 141. The wards are divided into specialised units (child, adolescent, eating disorder and psychotherapy wards), and biological wards, for adult patients with serious mental illness requiring medium- to long-term admission. There are three biological wards, comprising 80 beds. Referrals to Tara Hospital are received from various surrounding acute hospitals and clinics.

### Study population

All adult patients admitted to the biological wards over the 2-year study period, from 01 January 2018 to 31 December 2019, who received clozapine at any point during this admission were included in the study. No sampling technique was applied.

### Data collection

The wards’ admission registers were used to determine which patients were admitted to the biological wards over the 2-year study period and thereafter their files were accessed from the hospital’s registry department. The clinical notes and discharge summaries in all the files were reviewed to determine which patients met the inclusion criteria for the study and to capture data from those files. The following variables were collected on the data collection sheet: demographics, clinical information, prescription at discharge, documented clozapine-related side effects and details of discontinuation of clozapine, where applicable. In cases where notes were illegible or where the interpretation was not clear, data were noticed to have been omitted or unknown.

### Data analysis

Data from the data collection sheets were captured on Microsoft Excel and summarised using descriptive statistics. Further analysis was performed by a biostatistician using SAS Version 9.2. Frequencies and percentages were calculated for categorical data and means, and standard deviations (s.d.) or medians and percentiles were calculated for numerical data. The Shapiro–Wilk test was used to investigate whether numerical data followed a normal distribution. A significance level (α) of 0.05 was applied.

### Ethical considerations

This study was a retrospective review of patient files and clinical notes, and as such patients were not actively involved in the study. Approval of this research study was granted by the University of Witwatersrand Postgraduate Assessor Committee and ethics clearance was obtained from the University of Witwatersrand Human Research Ethics Committee (reference no. M210326-MED21-02-012). Institutional approval was obtained from Tara Hospital’s Research Committee and Chief Executive Officer. All patient information was kept confidential by allocation of study number to each patient, the list of which was kept in a locked cabinet on-site. Only the study numbers appeared as identifiers on the data collection sheet.

## Results

### Prevalence and indications for clozapine use

Of the 307 patients admitted to the biological wards over the study period, 102 met inclusion criteria, and thus the prevalence of clozapine use was 33.2%. Treatment-resistant psychosis was the most common indication for clozapine (98.0%, *n* = 100), with the remainder being for tardive dyskinesia (2.0%, *n* = 2). Regarding the number of documented previous failed antipsychotic trials, most patients (97.1%, *n* = 99) had a history of two or more failed antipsychotic trials prior to clozapine (2 trials, *n* = 51; 3 trials, *n* = 29; 4 trials, *n* = 17; 5 trials, *n* = 2).

### Demographics and clinical characteristics

The patients’ demographic and clinical characteristics are displayed in Table 1.

**TABLE 1:** Demographics and clinical characteristics of patients treated with clozapine in the biological wards at Tara Hospital during the study period.

Patient demographic and clinical variables (*n* = 102)	*n*	%
**Gender**		
Female	33	32.4
Male	69	67.6
**Ethnicity**		
Black people	91	89.2
Mixed race people	6	5.9
Caucasian people	3	2.9
Indian people	2	2.0
**DSM-5 diagnosis**		
Schizophrenia and other psychotic disorders:	100	98.0
*Schizophrenia*	55	53.9
*Schizoaffective disorder: bipolar type*	36	35.3
*Schizoaffective disorder: depressive type*	3	2.9
*Schizoaffective disorder: type unspecified*	1	1.0
*Psychotic disorder because of HIV*	2	2.0
*Psychotic disorder because of TBI*	1	1.0
*Schizophreniform disorder*	1	1.0
*Substance-induced psychotic disorder*	1	1.0
Bipolar 1 disorder	1	1.0
Major neurocognitive disorder because of HIV	1	1.0
**Pre-existing medical conditions**		
None	65	63.7
Presence of a pre-existing medical condition	36	35.3
*HIV*	11	10.8
*Hypertension*	8	7.8
*Dyslipidaemia*	6	5.9
*Hypothyroidism*	5	4.9
*Epilepsy*	2	2.0
*Diabetes mellitus: type 2*	2	2.0
*Diabetes mellitus: type 1*	1	1.0
*Chronic sinusitis*	3	2.9
*Traumatic brain injury*	2	2.0
*Rheumatoid arthritis*	2	2.0
*Chronic hepatitis B infection*	1	1.0
*Chronic kidney disease*	1	1.0
*Hepatitis A infection*	1	1.0
*Peri-anal warts*	1	1.0
*Previous neurosyphilis*	1	1.0
Unknown	1	1.0
**Cigarette smoking**		
Smoker†	49	48.0
Non-smoker	52	51.0
Unknown	1	1.0

Note: For the age in years: median = 37, range = 18–62 and interquartile range = 28–47.

HIV, human immunodeficiency virus; IQR, interquartile range; TBI, traumatic brain injury; DSM-5, diagnostic and statistical manual of mental disorder, fifth edition.

† , median cigarette pack year history of 3.6.

### Prescription characteristics

The total number of medications on discharge was high in both groups, those discharged on clozapine (mean 4.5; range 1–9) and those in whom clozapine was discontinued (average 4.1; range 1–8). According to the Chi-Square test, this was not significant (*p* = 0.1801). Details regarding co-prescribed psychotropic and non-psychotropic medications are shown in Table 2 and Table 3. The frequency of flupenthixol decanoate prescriptions was significantly higher in the clozapine-discontinued group as opposed to the discharged-on-clozapine group (64.3% vs. 30.7%; *p* = 0.0322) and similarly with regard to the frequency of anticholinergic prescriptions (35.7% vs. 11.4%; *p* = 0.0474). Of the 88 patients (86.3%) who remained on clozapine at discharge, the median total daily dose of clozapine was 350 mg, with an interquartile range (IQR) of 250 mg–450 mg, and maximum dose of 575 mg. Male clozapine doses did not follow a normal distribution, and as such, we report on the median dose as 225 mg, with an IQR of 125 mg–400 mg (maximum dose was 575 mg). Female clozapine doses did follow a normal distribution, with a mean dose of 260 mg, a range of 25 mg–575 mg, and s.d. of 155 mg. Almost half of the patients were on clozapine split-dosing schedules (*n* = 37; 42.0%), of which 7 were female (24.1%) and 30 were male (50.8%), which was statistically significant (Satterthwaite method Pr > |t| 0.0012).

**TABLE 2:** Psychotropic medications on discharge of patients treated with clozapine in the biological wards at Tara Hospital during the study period.

Medication	Discharged-on-clozapine (*n* = 88)		Clozapine-discontinued (*n* = 14)		*p*-value
	*n*	%	*n*	%	
**Antipsychotic**					
0 AP	0	0.0	1	7.1	-
1 AP	30	34.1	6	42.9	-
≥2 AP	58	65.9	7	50.0	-
Clozapine	*88*	*100.0*	*0*	*0.0*	-
Flupenthixol decanoate	*27*	*30.7*	*9*	*64.3*	***0.0322***
Amisulpride	*21*	*23.9*	*6*	*42.9*	*0.2418*
Zuclopenthixol decanoate	*8*	*9.1*	*2*	*14.3*	*0.8875*
Haloperidol	*2*	*2.3*	*0*	*0.0*	*0.6390*
Paliperidone palmitate	*2*	*2.3*	*0*	*0.0*	*0.6390*
Risperidone	*1*	*1.1*	*2*	*14.3*	*0.0640*
Olanzapine	*1*	*1.1*	*2*	*14.3*	*0.0640*
Aripiprazole	*1*	*1.1*	*0*	*0.0*	*0.2899*
**Mood stabiliser**					
0 MS	34	38.6	7	50.0	0.6101
≥1 MS	54	61.4	7	50.0	-
Sodium valproate	*46*	*52.3*	*6*	*42.9*	0.7184
Lithium	*14*	*15.9*	*2*	*14.3*	0.6468
Lamotrigine	*9*	*10.2*	*1*	*7.1*	0.8875
Carbamazepine	*1*	*1.1*	*0*	*0.0*	0.2899
**Antidepressant**					
0 AD	82	93.2	12	85.7	0.6629
1 AD	6	6.8	2	14.3	-
Fluoxetine	*3*	*3.4*	*1*	*7.1*	*0.9203*
Venlafaxine	*3*	*3.4*	*1*	*7.1*	*0.9203*
**Benzodiazepine**					
0 BZ	86	97.7	12	85.7	0.1583
1 BZ	2	2.3	2	14.3	-
Clonazepam	*1*	*1.1*	*1*	*7.1*	*0.6390*
Lorazepam	*1*	*1.1*	*1*	*7.1*	*0.6390*

Note: Data in italics are specific anti-psychotics, under the category of anti-psychotics (AP) and data in bold are statistically significant finding.

AD, antidepressant; AP, antipsychotic; BZ, benzodiazepine; MS, mood stabiliser.

**TABLE 3:** Co-prescribed non-psychotropic medications on discharge of patients treated with clozapine in the biological wards at Tara Hospital during the study period.

Medication	Discharged-on-clozapine patients (*n* = 88)		Clozapine-discontinued patients (*n* = 14)		*p*-value
	*n*	%	*n*	%	
Stool softener & laxative (Lactulose, Senokot, Fybogel and Liquid paraffin)	29	33.0	2	14.3	0.2713
Beta-blocker (Propranolol)	26	29.5	3	21.4	0.5598
Lipid-modifying agent (Simvastatin and Atorvastatin)	19	21.6	4	28.6	0.8065
Glucose-lowering agent (Metformin, Glimepiride and Actraphane)	11	12.5	1	7.1	0.8875
Vitamin supplement (Thiamine, Folate and Vitamin Bco)	9	10.2	3	21.4	0.4463
Antihypertensive (Amlodipine and Hydrochlorothiazide)	9	10.2	1	7.1	0.8875
Anticholinergic (Orphenadrine and Biperiden)	10	11.4	5	35.7	**0.0474**
HIV Antiretroviral (Efavirenz, Emtricitabine and Tenofovir)	9	10.2	2	14.3	1 0000
Gastro-oesophageal reflux disease and peptic ulcer treatment (Omeprazole)	7	8.0	0	0.0	0.5967
Urological (Oxybutynin and Tamsulosin)	6	6.8	0	0.0	0.6892
Thyroid hormone (Levothyroxine)	5	5.7	0	0.0	0.8065
Antihistamine (Cetirizine and Promethazine)	5	5.7	0	0.0	0.8065
Hormonal contraceptive (Combined oral contraceptive and Medroxyprogesterone acetate depot)†	4‡	13.8‡	1§	2.5§	0.8875
Anti-coagulant (Enoxaparin sodium and Warfarin)	1	1.1	0	0.0	0.2899
Disease modifying anti-rheumatic drug (DMARD) (Methotrexate and Chloroquine)	1	1.1	0	0.0	0.2899
Anti-inflammatory (Aspirin)	1	1.1	0	0.0	0.2899
Gastrointestinal antispasmodic agent (Hyoscine butylbromide)	1	1.1	0	0.0	0.2899

HIV, human immunodeficiency virus.

† , female patients (*n* = 33);

‡ , *n* = 29;

§ , *n* = 4.

### Side effects profiles

Side effects are shown in Table 4. The most frequently documented side effect of clozapine was weight gain, followed by tachycardia and constipation.

**TABLE 4:** Prevalence of clozapine-related side effects among patients in the biological wards at Tara Hospital during the study period who were discharged on clozapine (*n* = 88).

Side effect	Prevalence	
	*n*	%
Weight gain	70	79.5
Tachycardia	31	35.2
Constipation	31	35.2
Hypersalivation	20	22.7
Dyslipidaemia	15†	17.0†
Sedation	12	13.6
Nocturnal enuresis	9	10.2
Gastro-oesophageal reflux disease	7	8.0
Diabetes mellitus type 2	5†	5.7†
Hypertension	2†	2.3†
Neutropenia	2	2.3
Tremor	1	1.1
Agranulocytosis, fever, hypotension, myoclonus, nausea and seizures	0	0

† , newly diagnosed.

The mean weight gain by the end of admission among the clozapine-discontinued patients was 1.6 kg, compared with 9.3 kg among the discharged-on-clozapine patients. Discharged-on-clozapine patients had a median body mass index (BMI) of 24.1 and 28.0, at admission and discharge, respectively (increased by 3.4, IQR 25.3–32.9). Clozapine-discontinued patients had a median admission BMI of 25.9 and a median discharge BMI of 25.4 (IQR 23.4–31.4). Differences in BMI at discharge between groups were statistically significant (*p* = 0.0408).

Rates of antihypertensive prescription on discharge increased (i.e. hypertension diagnosed during current admission) only in the discharged-on-clozapine group, by two. Rates of lipid-lowering medications, indicative of dyslipidaemia, were increased in both populations of patients: increased twofold in the clozapine-discontinued group to a total of four patients, and more than fourfold increase in overall number in the discharged-on-clozapine group (total of 19 patients). This was statistically significant (*p* = 0.0282).

Two patients experienced neutropenia during their admission, with neutrophils at their lowest of 1.08 (range 1.40–4.20). Clozapine was not discontinued, and at discharge, neutrophils had normalised.

### Clozapine discontinuation

Clozapine was discontinued in 14 patients (13.7%). The mean dose at discontinuation was 246 mg, with an s.d. of 126 mg (*W* = 0.903379, *p* = 0.1263). The mean duration of therapy prior to discontinuation was 83.5 days, s.d. 38.8 days and range 21–140 days (*W* = 0.884554, *p* = 0.0822). Indications for clozapine discontinuation are shown in Table 5.

**TABLE 5:** Indications for discontinuation of clozapine (*n* = 14).

Indication	*n*	%
**Potentially life-threatening side effects**		
Ileus	1	7.1
Agranulocytosis (ANC < 0.5)	0	0.0
Myocarditis	0	0.0
Cardiomyopathy	0	0.0
Prolonged QTc interval (>500 ms)	0	0.0
Neuroleptic malignant syndrome	0	0.0
Venous thromboembolism	0	0.0
Hyperglycaemic emergency	0	0.0
Total	1	7.1
**Other potentially serious and/or intolerable side effects**		
Tachycardia	4	28.6
Neutropenia	3	21.4
Constipation	1	7.1
Nocturnal enuresis and hypersalivation	1	7.1
Worsening OCD symptoms	1	7.1
Total	10	71.3
**Other**		
No longer clinically indicated	1	7.1
No clinical response in the treatment of tardive dyskinesia	1	7.1
No clinical response in the treatment of psychosis with a combination of intolerable side effects: Constipation, hypersalivation, sedation and tachycardia	1	7.1
Total	3	21.3

ANC, absolute neutrophil count (× 10^9^/L); QTc, cardiac ventricular repolarisation; OCD, obsessive-compulsive disorder.

One patient experienced a potentially life-threatening side effect, ileus, which was successfully managed without surgery. Ten patients discontinued clozapine because of other potentially serious and intolerable side effects, or a combination of side effects and poor clinical response. Tachycardia was most common culprit for intolerability. Three of the patients did incur neutropenia, which resulted in clozapine discontinuation, but none met the criteria for agranulocytosis (< 0.5 × 10^9^/L).^7^ Neutropenia was confirmed on repeat testing for one of these patients, with an initial white cell count (WCC) of 3.49 and then 3.28, and ANC of 0.76 and then 0.92. In the second case, the WCC was 1.45 and the ANC 0.96. White cell count and/or absolute neutrophil count testing was not repeated prior to clozapine discontinuation in this patient. The last case involved an immunocompromised patient, who was human immunodeficiency virus (HIV) positive with a CD4 count of 340 (normal range 500 cells per mm^3^–1500 cells per mm^3^), and a declining neutrophil count during the early stage of clozapine initiation (dose of 75 mg). In this patient, the ANC dropped from 3.52 to 1.75, and this was deemed to be because of a combination of clozapine and co-trimoxazole (indicated for prophylaxis from opportunistic infections). Clozapine and co-trimoxazole were both discontinued and the ANC normalised. All clozapine-discontinued patients recovered and there were no clozapine-related mortalities.

## Discussion

A third (33.2%) of all the patients admitted to the biological wards at Tara Hospital over the study period received a trial of clozapine. The percentage of in-patients diagnosed with schizophrenia or other psychotic disorders that were treated with clozapine was not calculated for the purpose of this study, but rather the percentage of all the biological admissions, representing a patient population inclusive of all severe mental illnesses, not only psychotic disorders. Nonetheless, this result demonstrates that clozapine is not uncommonly used in the biological wards, which was not an unexpected finding as Tara Hospital is a medium to long-term treatment facility, likely to manage a greater proportion of patients who have responded poorly to standard first-line treatment and are treatment-resistant.

Treatment-resistant schizophrenia (or psychosis), defined as ‘the persistence of positive symptoms despite ≥ 2 trials of adequate dose and duration of antipsychotic medication with documented adherence’,^25^ was found to be the indication for clozapine use in almost all of the patients in this study. Furthermore, evidence of delayed initiation of clozapine was noticed, with 48% of the patients with treatment resistance having had between three and five documented antipsychotic trials prior to clozapine. Delays in the appropriate commencement of clozapine lead to poorer long-term outcomes.^26^ Barriers to clozapine use have been described in three categories: patient and medication-related, clinician-related and health system-related. Patient and medication-related barriers include non-compliance with blood testing schedules, intolerance of side effects and polypharmacy. Clinician-related barriers refer to a lack of knowledge and inexperience in identifying suitable clozapine candidates, prescribing clozapine and managing side effects, as well as the need for close monitoring and perceived poor adherence. Health system barriers refer to resource limitations and care continuity concerns regarding a more complex monitoring protocol. These barriers impact both clozapine initiation and continuation.^27^ However, in a study performed on psychiatric outpatients in KwaZulu-Natal (KZN), overall high rates of encouraging attitude and knowledge towards clozapine were reported by patients.^28^ Another study, conducted in Australia, compared the subjective experience of clozapine side effects and outcomes of clozapine-treated patients, with the perceived experience as assessed by their clinicians. In this study, stable clozapine users self-reported greater satisfaction with clozapine compared with what their clinicians believed them to be experiencing. Furthermore, only 19% of users reported disdain at blood tests, compared with the clinician estimate of 52%.^29^

Regarding the characteristics of clozapine users, the KZN study found a male and black race predominance of 67% and 71%.^28^ similar to the demographics of the population in this study (67.6% and 89.2%, respectively). Rates of pre-existing diagnoses of hypertension (7.8%) and diabetes mellitus type 1 and 2 (3%) in this study were lower compared with that of the general SA adult population, where hypertension rates of 44% (males) and 46% (females), and diabetes mellitus rates of 8% (males) and 13% (females) have been found.^30^ Likewise, with regard to pre-existing dyslipidaemia (5.9%) in this study. Little data exist on the prevalence of dyslipidaemia in the SA general population, but a study performed in Mpumalanga found a rate of 67%, however, with less than 1% being diagnosed and on treatment.^31^ These study findings were noteworthy, as not only higher rates of medical comorbidity (and reduced life expectancy) have been found in patients with schizophrenia but also increased risk of undetected (and inadequately treated) medical illness relative to the general population.^32^ Cigarette smoking was found to be highly prevalent in this study (48%) compared with the SA general population, where rates have been reported at 8% (females) and 37% (males),^30^ which was not a surprising finding, as smoking prevalence is known to be up to fivefold greater among patients with schizophrenia.^33^

High rates of polypharmacy were found across both groups in this study. Psychiatric polypharmacy, the prescription of two or more psychotropic medications, occurs commonly and can be described as same-class, multi-class, adjunctive, augmentation and total polypharmacy.^34^ Regarding antipsychotic polypharmacy specifically, this was common in both the discharged-on-clozapine and clozapine-discontinued groups (65.9% and 50.0%). In the discharged-on-clozapine group, the most common second antipsychotic was flupenthixol decanoate (30.7%), followed by amisulpride (23.9%). Despite foundational evidence behind clozapine combinations growing, it is not currently sufficient to delineate a treatment schedule. Although combination strategies may not lead to substantial clinical improvement, they can allow for clozapine dose reduction, and may thus benefit tolerability.^15^ A significantly greater use of flupenthixol decanoate was found in the clozapine-discontinued group (64.3%; *p* = 0.0322), compared with the discharged-on-clozapine group, which may also explain the significantly greater use of anticholinergics in the clozapine-discontinued group (*p* = 0.0474). This trend is echoed in a systematic review and meta-regression of global and regional trends of antipsychotic polypharmacy, which found antipsychotic polypharmacy significantly associated with the following: inpatient status, diagnosis of schizophrenia, greater long-acting injectable antipsychotic prescription and anticholinergics.^35^ There were no significant differences with regard to the use of other psychiatric and non-psychiatric medications between groups. The most frequently prescribed non-psychiatric medications were stool softeners or laxatives (33.0%), beta-blockers (29.5%) and lipid-lowering agents (21.8%) in the discharged-on-clozapine group and anticholinergics (34.7%), lipid-lowering agents (28.6%) and beta-blockers (21.4%) in the clozapine-discontinued group.

The most common side effects found in this study were weight gain, tachycardia and constipation. A recent UK-based data review of electronic health records of clozapine users from 2007 to 2016 similarly showed high rates of weight gain (56%), tachycardia (25%) and constipation (25%), with other notable side effects including hypersalivation (48%), sedation (46%) and insomnia (33%).^36^ As a result of the high rates of polypharmacy in this study, it was not possible to attribute side effects exclusively to clozapine. Aside from medications, other considerations are of multi-causative processes in the development of side effects such as dyslipidaemia and include sedentary lifestyle, poor diet, age and genetics.^37^ The discharged-on-clozapine patients had significantly greater weight gain, as well as increased rates of lipid-lowering agent initiations during the course of their admission. However, there was no significant difference between the overall rate of lipid-lowering prescriptions (i.e. for both pre-existing and newly diagnosed dyslipidaemias) in both groups. There were no cases of agranulocytosis, myocarditis, cardiomyopathy, prolonged QTc or clozapine-associated deaths in this population, all side effects, which are known to be rare.^8^ Regarding neutropenia, this occurred in two patients (2.3%) from the discharged-on-clozapine group, and three patients from the clozapine-discontinued group, accounting for 21.4% of clozapine discontinuations.

Consensus on the threshold for treatment interruption because of neutropenia is not conclusive. According to USA guidelines, the threshold is < 1.0 × 10^9^/L, or < 0.5 × 10^9^/L for patients with benign ethnic neutropenia (BEN), compared with the UK, which uses < 1.5 × 10^9^/L, or < 1 × 10^9^/L in cases of BEN.^38^ Benign ethnic neutropenia, which occurs in up to a third of individuals of African and Middle Eastern descent, is well-described and poses no increased risk for infection.^9,39^ Larger epidemiological studies have described BEN as an important contributor to under-prescription and early discontinuation of clozapine. Consequently, lower thresholds for clozapine initiation and discontinuation have been recommended in such patients.^39^ ‘Morning pseudoneutropenia’, caused by diurnal variation in circulating white blood cells, may also lead to unnecessary clozapine discontinuations.^11^

The rate of clozapine discontinuation in this study was relatively low (13.7%); however, only in-patient discontinuations were reviewed. A retrospective medication utilisation review, performed in the Eastern Cape, of a small cohort of patients discharged on clozapine found discontinuation rates of 29% and 35% at 3 and 6 months, respectively.^24^ Worsening of obsessive-compulsive symptoms, found in one clozapine-discontinued patient in this study, is a rare side effect that has been found to be associated with clozapine, as well as with other atypical antipsychotics.^40^ Only one patient had a potentially life-threatening side effect, namely ileus, which was resolved upon medical management and discontinuation of clozapine. There were no fatalities. Clozapine doses in this study were all less than the maximum licenced dose for clozapine.^15^

### Strengths and limitations

This study not only highlighted the issue of delays in clozapine initiation, polypharmacy and the side effect burden among clozapine users but also the relatively low discontinuation rate. Limitations because of the study’s retrospective nature included missing files and incomplete clinical notes. This study did not account for clozapine side effects that may have occurred and subsequently resolved during admission, or any that were not otherwise documented in the discharge summary. No side effect rating scales were used. Polypharmacy and comorbid conditions made interpreting side effects attributable only to clozapine difficult. There was no comparison with regard to the length of stay between the two groups of patients. Furthermore, the results from this study may not be generalisable to other settings as Tara Hospital is a medium- to long-term specialised psychiatric unit.

### Implications and recommendations

Proper monitoring and pre-emptive management of clozapine-related side effects, particularly in the first few months of treatment, is recommended. This may also assist in reducing avoidable or unnecessary clozapine discontinuations.^41^ The use of side effect rating scales, for example, the Glasgow Antipsychotic Side-Effects Scale for Clozapine (GASS-C),^42^ as part of routine clinical care is also recommended. Metabolic effects, especially weight gain was common. Lifestyle interventions such as dietary changes and increased physical activity are thus also important.^43^ Regular medication review is advised to re-evaluate indications for polypharmacy as patients’ symptoms and side effects may attenuate.^34^ There is a need for further prospective research in SA regarding the long-term health and psychiatric outcomes of clozapine-treated psychiatric patients, including comparisons specifically to clozapine-discontinued patients, as well as those with treatment-resistant psychosis receiving other antipsychotic medications. Combination antipsychotic therapy has become commonplace and is a field of great potential, but its risks and benefits need to be substantiated by further research. Research related to patterns of clozapine use, side effects and management thereof, as well as clozapine monitoring protocols would be beneficial. Exploration of the potential usefulness of plasma clozapine levels in our setting would also be valuable, as would research into South African clinicians’ attitudes to clozapine prescription, and their perceived barriers to its use. In addition, studies regarding the pharmacoeconomics of clozapine use among the population of patients with treatment-resistant schizophrenia may be of interest to both mental healthcare providers and policymakers.
